# A Priori Information Based Time-Resolved 3D Analysis of the Trajectory and Spatial Orientation of Fast-Moving Objects Using High-Speed Flash X-ray Imaging

**DOI:** 10.3390/jimaging8020028

**Published:** 2022-01-28

**Authors:** Ralph Langkemper, Stefan Moser, Markus Büttner, Dominik Rakus, Axel Sättler, Siegfried Nau

**Affiliations:** Fraunhofer Institute for High-Speed Dynamics, Ernst-Mach-Institut, EMI, Ernst-Zermelo-Straße 4, 79104 Freiburg, Germany; stefan.moser@emi.fraunhofer.de (S.M.); markus.buettner@emi.fraunhofer.de (M.B.); dominik.rakus@emi.fraunhofer.de (D.R.); axel.saettler@emi.fraunhofer.de (A.S.); siegfried.nau@emi.fraunhofer.de (S.N.)

**Keywords:** 2D to 3D registration, high-speed X-ray imaging, fast-moving object, sabot discard, a priori information

## Abstract

This paper shows that the X-ray analysis method known from the medical field, using a priori information, can provide a lot more information than the common analysis for high-speed experiments. Via spatial registration of known 3D shapes with the help of 2D X-ray images, it is possible to derive the spatial position and orientation of the examined parts. The method was demonstrated on the example of the sabot discard of a subcaliber projectile. The velocity of the examined object amounts up to 1600 m/s. As a priori information, the geometry of the experimental setup and the shape of the projectile and sabot parts were used. The setup includes four different positions or points in time to examine the behavior over time. It was possible to place the parts within a spatial accuracy of 0.85 mm (standard deviation), respectively 1.7 mm for 95% of the errors within this range. The error is mainly influenced by the accuracy of the experimental setup and the tagging of the feature points on the X-ray images.

## 1. Introduction

The observation and analysis of highly dynamic processes are of interest in a broad range of research fields, ranging from car-crash investigations (e.g., References [1,2]) and ballistics processes (e.g., References [3,4]) to geological impact (e.g., Reference [5]) and impacts of space debris on satellites (e.g., Reference [6]). As these processes happen on a timescale of milliseconds down to microseconds [7], both experimental observation and analysis are challenging.

As a result of hot gases, dust, debris, luminous effects or overlapping objects, it is not always possible to observe it by using high-speed cameras. The same applies for processes within objects.

Therefore, a feasible approach for these cases is using X-ray technology. The state-of-the-art for X-ray analysis of dynamic processes is imaging by using flash X-ray tubes combined with a suitable detector, resulting in X-ray images with an exposure time of 20 to 35 ns [8,9]. Depending on the application, the two-dimensional information gained from the X-ray image is sufficient. However, if spatial information for all three dimensions is needed, the task is a lot more challenging. The means of choice for static objects or slower processes is computed tomography (CT), using several hundred X-ray projections to reconstruct a 3D volume. With certain limitations, this approach is also applicable for fast dynamic processes. A method for high-speed tomography is shown in References [10,11,12], using only six X-ray projections, resulting in a three-dimensional model at one certain point in time. A similar and additionally time-resolved approach based on theoretical simulations is described in Reference [13]; however, this method has not been tested experimentally within this paper. Both methods work without any knowledge about the tracked object. Contrary to this, the shape of the observed objects in the example used in this paper is known and available.

Using the known shape of the observed objects as a priori information, we propose an approach using only two flash X-ray projections per time step, facing the objects from different viewing angles to derive their position and orientation in a highly dynamic process. In order to determine a trajectory, the 3D positions and orientations are determined in the same way at several time steps. We tested the proposed method for the tracking of fast objects in three dimensions, using the example of the sabot discard of a laboratory projectile.

Several decades ago, similar approaches were made in the field of medical radiology in which the placement of a patient or a body part is spatially located in all degrees of freedom, using X-ray projections or tomographic slices [14,15,16,17]. In the following years, several scientific papers were published, in which either 3D/2D registration methods for image-guided interventions were developed or validated [18]. Similar to the approach in this paper, a 3D model, usually an anatomical structure generated via computed tomography or magnet resonance tomography, is registered with the help of X-ray images. Normally, two X-ray images with a perpendicular angle of perspective are used, but there are also approaches that use only one X-ray image [14,19]. The easiest method for the registration is the usage of medical landmarks or feature points that are either manually or automatically picked on the X-ray images. Besides feature-point-based registration, other papers alternatively show intensity- or gradient-based methods for the registration of the model [18].

A very similar solution is made for motion analysis of animals [20,21,22] by using an “X-Ray reconstruction of Moving Morphology (XROMM)” [23]. This method uses a model of the bone as a priori information and registers the model by two X-ray videos from two different directions.

To the knowledge of the authors, these methods have not been used to investigate the orientation and position of objects traveling with high-speed. Due to the fact that, in this paper, a fast-moving object is examined, the experimental setup has to be adjusted to the associated challenges and differs essentially from the solution for the data acquisition in the medical and biological field (XROMM). To examine the behavior of the object over time, the experimental setup that is described in Section 2 was developed. The registration method in this paper is a manually picked feature-point-based approach.

## 2. Materials and Methods

### 2.1. Experimental Setup

**Figure 1:**
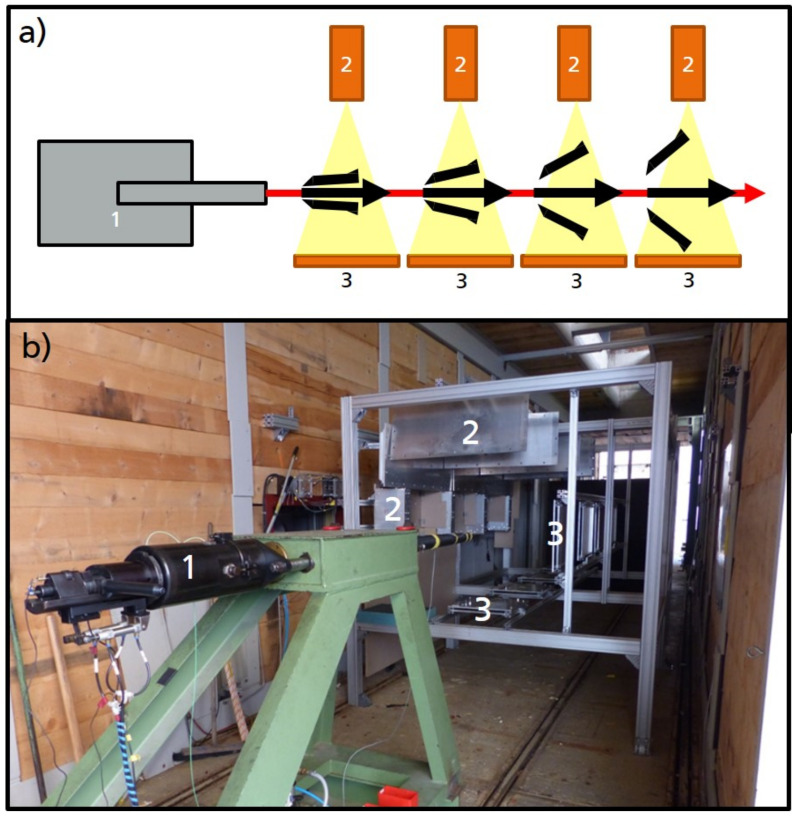
Experimental setup: accelerator—in this case, a 30 mm smooth bore cannon (1); flash X-ray tubes (2); and image plates (3). (**a**) Schematic drawing of the experimental setup showing the four imaging channels (consisting of one flash X-ray source and one detector each) of one of the two perpendicular viewing directions. (**b**) Photo of the setup. Only the X-ray tubes and image plates of position 1 are marked.

To examine the sabot discard from a projectile, X-ray images were recorded at four points in time. X-ray pair setups were positioned at 500, 1500, 2500 and 3500 mm after the muzzle of the accelerator tube. As an accelerator device, a 30 mm smooth bore powder cannon was used, delivering a non-spinning projectile with velocities of up to 1600 m/s. For each position, two X-ray images were acquired from two different perspectives perpendicular to the firing axis and each other, similar to the shown X-ray setup in Reference [18]. The setup is shown in Figure 1.

The X-ray setup consisted of 150 kV flash X-ray sources [8], with a focal-spot size of about 1 mm and detectors composed of image plates placed in cartridges with a resolution of 100 µm. The accuracy of the position of the image plates within the cartridges was in the range of several millimeters and a possible source of error. To protect the X-ray equipment from the blast of the accelerator, it was covered with 10 mm aluminum plates on each side. A rough estimation that used the Beer–Lambert law indicates a reduction for 20 mm aluminum of about 50% of the photons that are utilizable for imaging, assuming the peak of the spectrum of the tube is around 80 keV. The distance between source and detector is 1.24 m and between source and firing axis is 0.72 m, resulting in a magnification factor of 1.72 and a detail discrimination around 1 mm. The X-ray tubes were triggered at defined points in time, depending on the velocity of the projectile. Due to the short exposure time (20–35 ns) the motion blur is neglectable. For an object traveling with 1600 m/s, it would be around 50 µm.

The behavior of the parts during the separation process is mostly determined by the aerodynamic processes and their internal mass distribution. All flying objects are assumed to be rigid bodies, as is consistent with the experimental X-ray images. To ensure that there is no influence on the aerodynamic behavior of the projectile and the sabot parts, it was not possible to place X-ray markers at these parts.

The experimental setup was constructed as a digital CAD model and physically built based on this model. To assure that the digital model and physical experimental setup matched adequately, relevant distances of the experimental setup were measured manually with a laser distance measurement, and with these measurements, the digital model was adapted to fit the physical setup more accurately. This adjusted digital model was used within the X-ray analysis as the representation of the experimental setup. Note that, for the method to be applicable, it is not necessary to achieve the exact positioning of the setup according to the (initial) CAD models. Only the knowledge of the physical positions of the setup used is important in order to have an accurate representation of the experimental setup.

**Figure 2:**
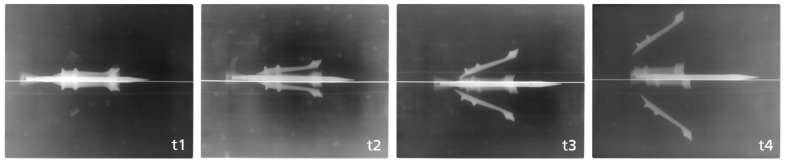
Example of the X-ray images for one orientation and all four positions (t1–t4). The first image at the point of time t1 is positioned 500 mm behind the muzzle; hence, t2 is at 1500 mm, t3 at 2500 mm and t4 at 3500 mm.

This geometric calibration process leaves a possible residue deviation between the CAD model and real experimental setup of several millimeters for the setup geometry for opposing ends of the ~4 m setup. For future experiments, we plan to use a laser tracker device, thus enabling very precise position measurements with an accuracy of a few ten micrometers over distances of several meters. Despite the use of precision-measuring devices, determining the exact focal-spot position of the X-ray sources and the precise position of the detectors is challenging. Therefore, a reference wire was included in the setup for calibration. It can be seen as a white horizontal line in Figure 2.

### 2.2. X-ray Analysis

The X-ray images (Figure 2) were analyzed, and the trajectory and orientation of the single parts were reconstructed with a software written especially for this purpose.

**Figure 3:**
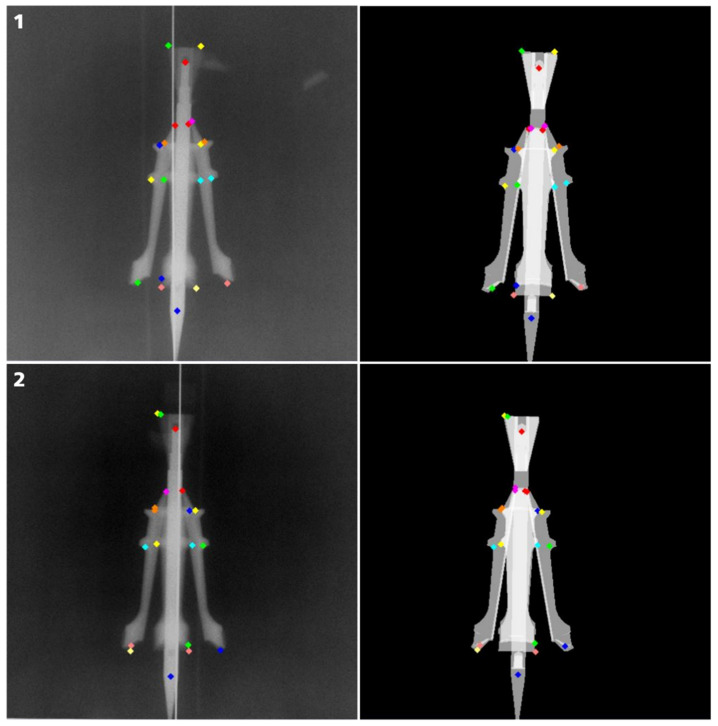
Example of the software-based X-ray analysis. On the left side, the X-ray images, including the set of tagged feature points, are displayed for one position (point in time) and two corresponding perspectives (1, vertical view; 2, horizontal view). The resulting positioning of the models is shown as a simulated X-ray image on the right side.

The geometry of the experimental setup and the CAD models of the projectile and sabot parts were used in the analysis software as a priori information. In a first step, the experimental setup was digitally replicated within the software; the 3D models of the parts of interest were imported into the software and manually placed at the approximate coordinates and orientations they were expected, and the X-ray images were imported. The more accurate the alignment between the digital representation and the physical experimental setup is, the more precise the results will be. In the next step, the position and orientation of the projectile and the sabot parts were reconstructed from the recorded X-ray images via the following steps:

1. Several feature points were defined at distinctive structures of the 3D model for each part. To assure a clear assignment for each feature point, the feature points were displayed in different colors, as one can see in Figure 3.
2. The positions of the feature points were tagged on the X-ray images manually. The accuracy of the tag on the X-ray image was mainly influenced by the image quality. Some structures were not visible in both X-ray images of a single position, due to the overlap of some parts on the projection. For these structures, the feature points may only be tagged in one of the X-ray images. Due to the overlap of the parts and the limited image quality mainly caused by the limited tube voltage of the flash X-ray sources used, the tags have to be set manually by experienced users. Additionally, the 3D shape of the parts must be taken into consideration, meaning a corner on the 3D model is not necessarily shown as a tip on the X-ray image.
  In future experiments the image quality could be optimized and the feature points may be detected automatically.
3. The “best fit” orientation is calculated by using an error-minimizing fitting method to find the position and orientation of the part in 3D. The best position is assumed to return the least square error sum of the distances of features selected in 3D on the model and the rays connecting the X-ray source and the projected position of the feature in 2D.
  To assist the user in the tagging process and to control the precision of the result by visual comparison, the software is able to calculate simulated radiographs of the scene and produces overlay images of the real and the simulated radiographs.

For this analysis, it is assumed that the parts are rigid bodies during the sabot discard. This assumption is backed by the X-ray images.

An example of this method is shown for one position in Figure 3. It shows for one point in time and for both imaging directions the respective X-ray image with the tagged feature points on the left side; and the right side shows a simulated X-ray image of the projectile with four sabot parts, including their feature points orientated in space with the same imaging geometry, such as the actual X-ray image. As a result, the examined objects—in this example, the projectile and sabot parts—can be exported as mesh models that are orientated and positioned within the coordinate system of the experimental setup. In this example, a right-hand-side coordinate system was used in which the origin is located at the muzzle of the accelerator, the *x*-axis is the firing axis, the *y*-axis is the horizontal and the *z*-axis is the vertical axis.

## 3. Results

**Figure 4:**
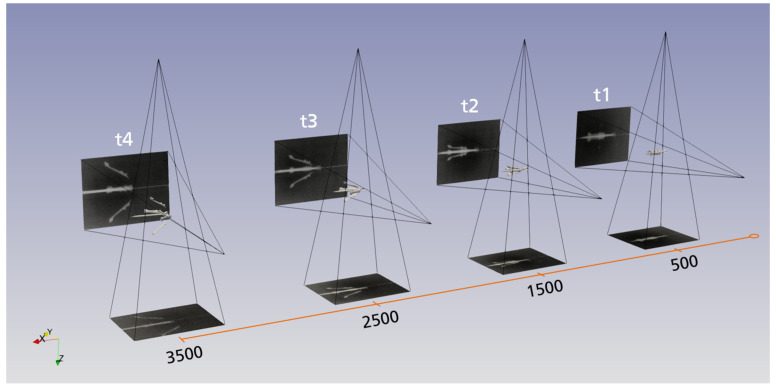
Illustration of the results of the evaluation software. The projectile, the sabot parts, the X-ray tubes and image plates are located and orientated in space in a single coordinate system. The firing axis is chosen as the *x*-axis, the *y*-axis is the horizontal and the *z*-axis is the vertical axis.

The result of the evaluation software is given as a 3D model of the projectile and its sabot parts placed within the coordinate system chosen for the experimental setup. Figure 4 shows the resulting scene for four points in time. The four measurement locations (i.e., four points in time) placed along the firing axis are shown together with the reconstructed respective sabot and projectile location and orientation. Included in Figure 4 are the positions of the flash X-ray sources and their corresponding image plates as the imaging system. It is observable that, as expected, the sabot separates after leaving the muzzle, increasing the distance and angle of its parts to the projectile over time.

**Figure 5:**
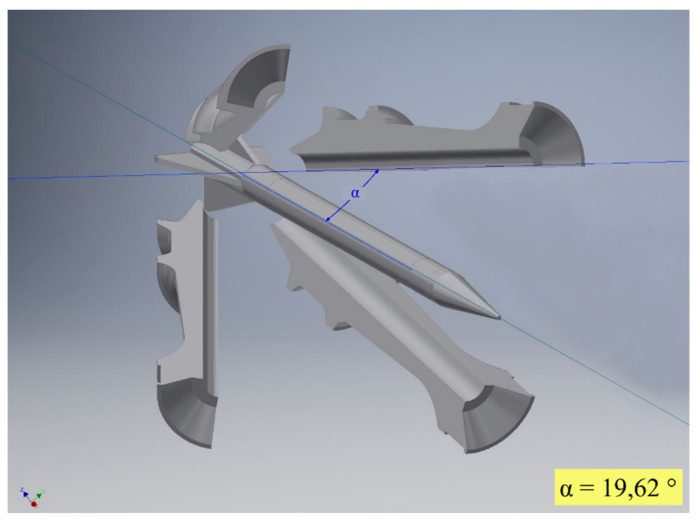
As a utility example, a close-up view of position 3 (t3) is shown. The angle between the projectile and one sabot part is measured. The exported meshes can be examined with various different software solutions.

These data can be used to examine the behavior of the sabot parts and their influence on the projectile movement. For evaluation purposes, each part can be exported for each time step in a standard 3D format, such as STL, including orientation and position, allowing the import into a variety of commercial 3D evaluation software programs. As an example, Figure 5 shows a close look at one single position in a CAD software program, including the measurement of an angle between the projectile and a sabot part. Further examination, including measuring different angles and distances, is easily possible.

It is important to point out that the analyzed object is non-spinning projectile, and the distinct assignment of all parts is possible for all time steps. For this configuration, it is possible to interpolate the overall movement of the analyzed parts. This is not necessarily achievable if the analyzed objects are spinning or moving in an atypical and abrupt way. For these cases, it has to be ensured that the frame rate is high enough to represent these events to prevent subsampling and aliasing effects.

## 4. Error Estimation and Discussion

It is not possible to calculate the error of the results directly, as there are several unknown sources of inaccuracy affecting this method. One of the main factors is expected to be the deviation between the digital representation used for the evaluation and the actual experimental setup. To minimize this error, the experimental setup was built with high accuracy. The error is expected to be in an order of magnitude of a few millimeters. Another relevant part is the error of the tagged feature points on the X-ray image. Due to blurring effects, e.g., the focal spot size, the low signal-to-noise ratio and the precision when tagging a feature point are estimated to be around ±1.5 mm in the image plane. If the parts of the objects that carry the feature points are overlapping, this error is estimated to grow by a factor of two to three.

**Figure 6:**
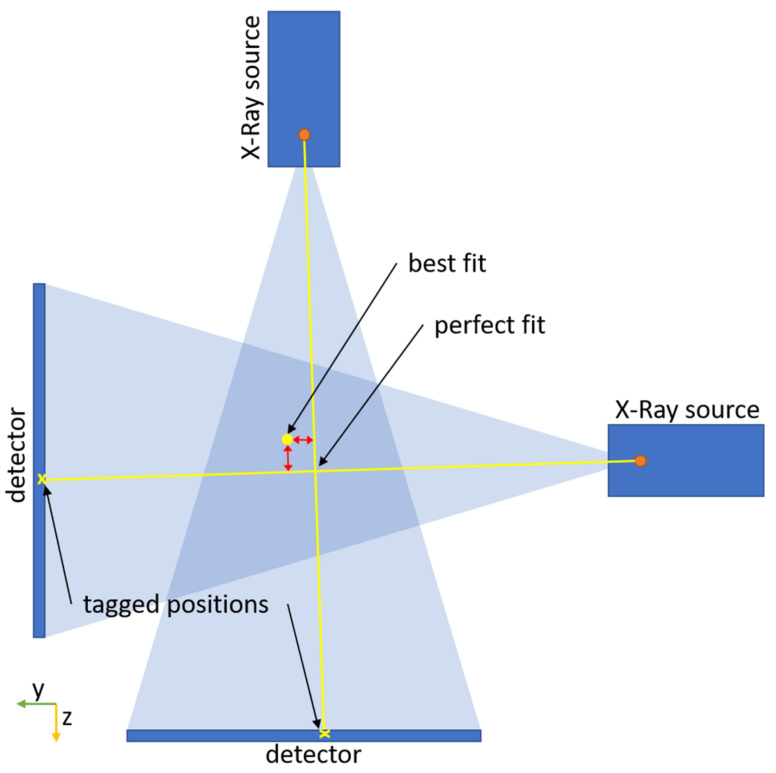
Schematic representation of the deviation for the error estimation in 2D (yz-plane). The deviation is shown as red arrows. The “perfect fit” is the intersection of the line between focal spot of the X-ray source and the tagged position on the X-ray image.

To estimate the error, four exemplary experiments were subjected to an in-depth statistical analysis, using all 626 tagged feature points. For each feature point, the deviation between its spatial position on the 3D model and its “perfect fit” for the tagged position on the X-ray images is derived for the *x*-, *y*- and *z*-axes. Figure 6 illustrates the deviation of a feature point between its “best fit” and “perfect fit” positions.

**Figure 7:**
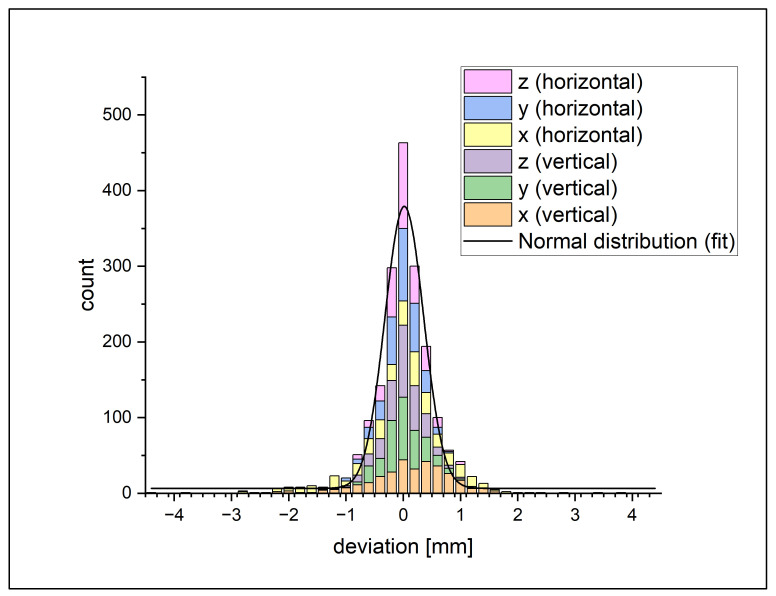
Histograms of the deviation between the feature points on the 3D model and the tagged position on the X-ray image. It shows the counts for a range of 0.2 mm. The colors show the results for each axis on the horizontal and vertical setup.

For the best fit, the location of the 3D feature points relative to each other is constrained by belonging to the same rigid body. Therefore, all discussed errors usually prevent a “perfect” fit (positioning of the rigid body) wherein all 3D feature points are projected onto the exact location where they are visible in the images. The effect of the errors is divided by the feature points by using a least-squares-minimization approach. To estimate the “perfect fit” position, the feature points in 3D were fitted without the constraints of the rigid 3D model. This led to a shift of the 3D feature points in space, given by the shortest distance in space they need to be moved to be projected onto the exact location at which they are observed in the images. This distance is regarded as the positioning error of singular 3D feature points and used for error analysis in the following. The histogram of all deviation values is shown in Figure 7.

As seen in the histogram, the data approximately show a typical normal distribution. Because of this, it is assumed that there is no notable systematic error. Taking a closer look, we see that the data of the single axis disclose that the curves of both *x*-axes have a lower amplitude and are wider than the other axes, meaning that the error on the *x*-axis (the firing axis) is significantly higher than the error of the other axes. Nearly all error values over 1 mm are on one of the *x*-axes. Consequently, the counts on the smaller errors are higher on the *y*- and *z*-axes. This is confirmed by the standard deviation seen in Table 1.

**Figure 8:**
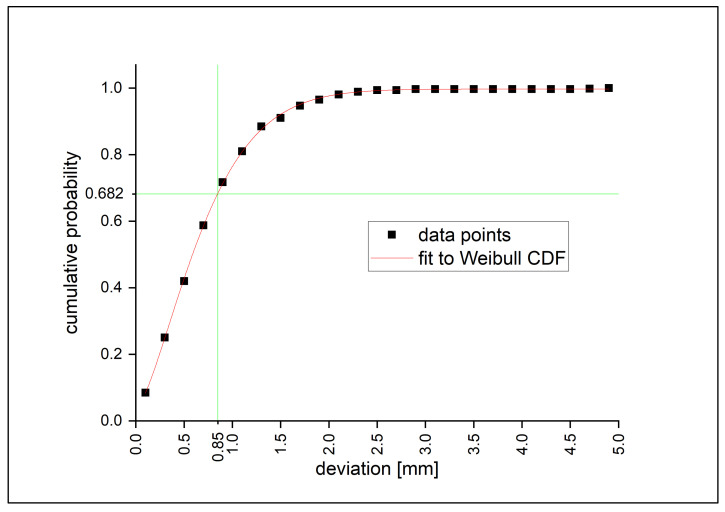
Cumulative probability plot of the spatial deviation (vector magnitude of x-, y- and z-deviation) of the feature points. The red line shows a Weibull cumulative distribution function (CDF) fit to these data points and is used to determine the standard deviation of the spatial deviation (shown in green).

To get the value of the spatial error, the magnitude of the *x*-, *y*- and *z*-error of the feature points is calculated. The results for all feature points are shown in Figure 8 as a cumulative plot.

The data show a typical cumulative Weibull distribution. To compare it with the standard deviation, the deviation value of 68.2% of the errors is derived for each experiment, here also referred to as standard deviation. These results are shown in Table 1.

As seen in Table 1, the error in *x*-direction is significantly higher than in *y*- and *z*-directions. While there is no obvious explanation for this biasing of the errors, some possible influences can be derived and will be investigated in the next set of experiments. One possible influence may be the nature of the placement methods of the image plates. They were placed within cartridges in which the image plates have a small backlash. It seems that the backlash is higher in the loading direction, which is orientated in the *x*-direction for both the horizontal and the vertical detector setup.

The axis deviations between the experiments vary a little: less for vertical geometry and more for horizontal. Again, this could be caused by the backlash of the image plates’ placement, but also by the small deformation of the experimental setup between the experiments, due to the muzzle blast.

There was no significant difference for the relative deviations of the projectile and sabot parts observable. For the spatial error, the standard deviation is quite similar for all experiments.

The standard deviation of all experiments is 0.85 mm; for 95% of the errors within the range, the error is 1.7 mm. This accuracy is roughly in the same order of magnitude to the early approaches within the medical field [16,19,24]

## 5. Conclusions and Outlook

Within the scope of this paper, an X-ray analysis method using a priori information was successfully adjusted and applied to the area of high-speed dynamics. It was shown that, via spatial registration of known 3D shapes with the help of 2D X-ray images, it is possible to derive the spatial position and orientation of the examined parts. Using four positions at four points of time, the time-dependent behavior can be examined.

A detailed error estimation shows that the achieved accuracy is comparable to the early approaches of similar methods within the medical field. The most relevant error factors are as follows:

- Deviation of experimental setup and its digital representation,
- Backlash of the image plate within the cartridges,
- Focal spot size of the X-ray source,
- Accuracy of the tagged feature points on the X-ray image.

To increase the accuracy in future experiments, the following steps can be realized:

To reduce the deviation between the experimental setup and its digital representation, an exact measurement of the experimental setup, using a laser tracker system capable of measuring each position and orientation of all relevant parts in space with an accuracy of better than 1 mm, should be used.

The backlash of the image plates within the cartridges can be reduced via prepossessing of the X-ray images with the help of X-ray markers with known positions that are displayed in the X-ray images.

## Figures and Tables

**Table 1:** Standard deviation of the deviation for *x*, *y*, *z* and the vector length for each experiment and detector geometry.

Standard Deviation (1/mm)	Vertical Geometry				Horizontal Geometry				Overall
	*x*	*y*	*z*	Norm (*x*, *y*, *z*) ^1^	*x*	*y*	*z*	Norm (*x*, *y*, *z*) ^1^	Norm (*x*, *y*, *z*) ^1^
Experiment 1	0.79	0.32	0.33	0.84	0.87	0.26	0.22	0.88	0.86
Experiment 2	0.50	0.31	0.31	0.70	0.80	0.32	0.29	0.84	0.77
Experiment 3	0.66	0.44	0.42	0.89	1.02	0.55	0.41	0.98	0.93
Experiment 4	0.60	0.34	0.34	0.74	0.92	0.59	0.62	1.01	0.87
All experiments	0.69	0.36	0.36	0.79	0.93	0.46	0.42	0.93	0.85

^1^ For 68.2% of the error values.

## Data Availability

Detailed data are confidential.
